# At Omaha

**Published:** 1898-08

**Authors:** 


### EDITORIAL.

### AT OMAHA.

The thirty-fifth annual meeting of the U. S. V. M. A. will direct association influences in an entirely new territory. It will reach a large number of the profession who for five or more years have felt the blighting influences of the depression of livestock and agricultural pursuits. These conditions have made the employment of members of the profession through the Central West meagre in character, and brought such uncertain and precarious incomes to the profession in general as to make existence only possible. With such conditions few could attend our meetings during this trying period, and slight was the incentive for self-improvement when the depressed livestock interests would not warrant the employment of professional skill. With the brighter outlook, the more favored condition of the future market for horses, the decline of the cycle craze, the uselessness beyond that of a toy of the automobiles, the future becomes again promising and auspicious. The greater interest in veterinary sanitary science, the subjects of meat- and milk-inspection, and the foreign exportation of our food and manufactured products will all tend to increased interest in the national association affairs, and be reflected in the increased attendance of many who have heretofore been denied this privilege. This is the last call, and every veterinarian in the Central West should take part in the Omaha meeting.

